# Neural Regulation of Interactions Between Group 2 Innate Lymphoid Cells and Pulmonary Immune Cells

**DOI:** 10.3389/fimmu.2020.576929

**Published:** 2020-10-29

**Authors:** Weiwei Chen, Qiang Shu, Jie Fan

**Affiliations:** ^1^ Department of Surgery, University of Pittsburgh School of Medicine, Pittsburgh, PA, United States; ^2^ The Children’s Hospital, Zhejiang University School of Medicine, National Clinical Research Center for Child Health, Hangzhou, China; ^3^ Research and Development, Veterans Affairs Pittsburgh Healthcare System, Pittsburgh, PA, United States; ^4^ McGowan Institute for Regenerative Medicine, University of Pittsburgh, Pittsburgh, PA, United States

**Keywords:** neuroimmunity, adaptive immunity, innate immunity, lung, group 2 innate lymphoid cells (ILC2)

## Abstract

Emerging evidence supports the involvement of nervous system in the regulation of immune responses. Group 2 innate lymphoid cells (ILC2), which function as a crucial bridge between innate and adaptive immunity, are present in large numbers in barrier tissues. Neuropeptides and neurotransmitters have been found to participate in the regulation of ILC2, adding a new dimension to neuroimmunity. However, a comprehensive and detailed overview of the mechanisms of neural regulation of ILC2, associated with previous findings and prospects for future research, is still lacking. In this review, we compile existing information that supports neurons as yet poorly understood regulators of ILC2 in the field of lung innate and adaptive immunity, focusing on neural regulation of the interaction between ILC2 and pulmonary immune cells.

## Introduction

The past decade has witnessed an unprecedented interest in the neural modulation of immunity (1–4). The immune barrier consists of innate and adaptive components that adopt different strategies to perceive and respond to pathogen challenge. In the context of innate immunity, innate lymphoid cells (ILCs) have been demonstrated to be a crucial bridge between both immunity branches (5, 6).

ILCs are a heterogeneous family of lymphocytes that lack re-arranged antigen receptors present on B and T cells. In earlier studies, lymphoid tissue inducer (LTi) cells and natural killer (NK) cells were initially identified as the subgroups of the ILCs (7–10). In recent years, more subgroups of the ILCs were discovered and, based on the surface markers, cytokines, and transcription factors, categorized into three major types, ILC1, ILC2 and ILC3 (11, 12). These ILCs groups have distinct phenotypic, developmental, and functional properties. They are the innate counterparts of T lymphocytes: ILC1, ILC2, and ILC3 mirror CD4^+^ T helper (Th)1, Th2, and Th17 cells, respectively, based on cytokine secretion and transcription factor expression (11, 13, 14). ILC1 consists of conventional NK cells and ILC1s. T-bet, a T-box transcription factor, is indispensable for the differentiation and interferon-gamma (IFN-γ) secretion ability of ILC1. RORα and GATA3 are essential for the development of ILC2, which can be grouped into transient, circulating inflammatory ILC2 (iILC2) and tissue-resident natural ILC2 (nILC2) types (15, 16). ILC3 comprises the classical lymphoid inducer (LTi) cells and LTi-like ILC3 with or without natural cytotoxicity receptors (NCRs), all of which rely on the RORγt (transcription factor) for development and secrete IL-17 and/or IL-22. ILCs protect individuals against infectious agents, response to inflammatory stimuli, and orchestrate lymphoid organogenesis and tissue repair, at various tissues especially mucosal barriers (17–19).

Among all subsets, ILC2 are the center of numerous investigations. They are mainly localized at mucosal barriers, e.g. the small intestine, skin, and lung (19–21). ILC2 are a master regulator of immune and inflammatory responses, but their own regulatory mechanisms remain largely elusive.

ILC2 are activated by host-derived alarmins such as IL-25, IL-33, and thymic stromal lymphopoietin (TSLP), which are expressed during tissue injury (22–24). Once activation takes place, ILC2 release large quantities of cytokines such as IL-4, IL-5, IL-6, IL-9, IL-10, IL-13, IL-17, and amphiregulin (16, 25–28). Furthermore, ILC2 interact with other cells through surface-bound molecules, such as CD80/86, MHC class II, PD-L1, OX40L, and inducible costimulator ligand (ICOS-L), and participate in immune-regulatory functions (29–32). ILC2 play critical roles in the regulation of inflammation, allergic immunity, metabolic homeostasis, parasite rejection, and tissue repair. Dysregulation of ILC2 contributes to inflammatory responses, including allergen-induced lung inflammation (33, 34), airway hyperreactivity (35), and atopic dermatitis (36).

Currently, the nervous system is found to have complex dual functions to quickly stimulate or suppress immune cells to defend the body against various inflammatory responses. There are continuing advances in our knowledge of neural regulation of ILC2, these brilliant results provide a new dimension of immune regulation (37–47). Studies have shown that receptors for norepinephrine, acetylcholine (Ach), neuromedin U (NMU), neuromedin B (NMB), α-Calcitonin Gene-Related Peptide (CGRP), and other neurotransmitters are present on T cells, dendritic cells (DCs), macrophages, ILC2, and other immune cells (19, 37, 38, 44, 48–50), and pattern-recognition receptors (PRRs) and cytokine receptors are distributed on neurons (51–54). Interestingly, immune cells are also able to synthesize and secrete catecholamines, acetylcholine, CGRP, and other neurotransmitters (39, 48, 49, 55). Moreover, ILC subtypes express the nicotinic and muscarinic cholinergic receptor for Ach, β_2_-adrenergic receptor (β_2_AR) for epinephrine and norepinephrine, calcitonin receptor-like (CALCRL) for CGRP, neuromedin U receptor 1 (NMUR1) for NMU, neuromedin B receptor (NMBR) for NMB, and VPAC1/2 (vasoactive intestinal peptide receptor) for vasoactive intestinal peptide (VIP) (19, 39, 44, 50, 56). These findings suggest physical machinery for neuro-immune communications. Also, type I cytokines can also influence cells of the center nervous system (CNS) and mediate what is called “sickness behavior” (57, 58). In this review, we highlight existing information that describes neurons as novel regulators of ILC2 in the context of pulmonary innate and adaptive immunity.

## Mechanisms Underlying ILC2 Interact with Other Immune Cells

ILC2 function both as initiator of adaptive immunity or as responder to signals produced by B and T cells. Using ILC2-targeted models, investigations have shown multiple mechanisms by which ILC2 regulate innate and adaptive immune system.

**Figure 1:**
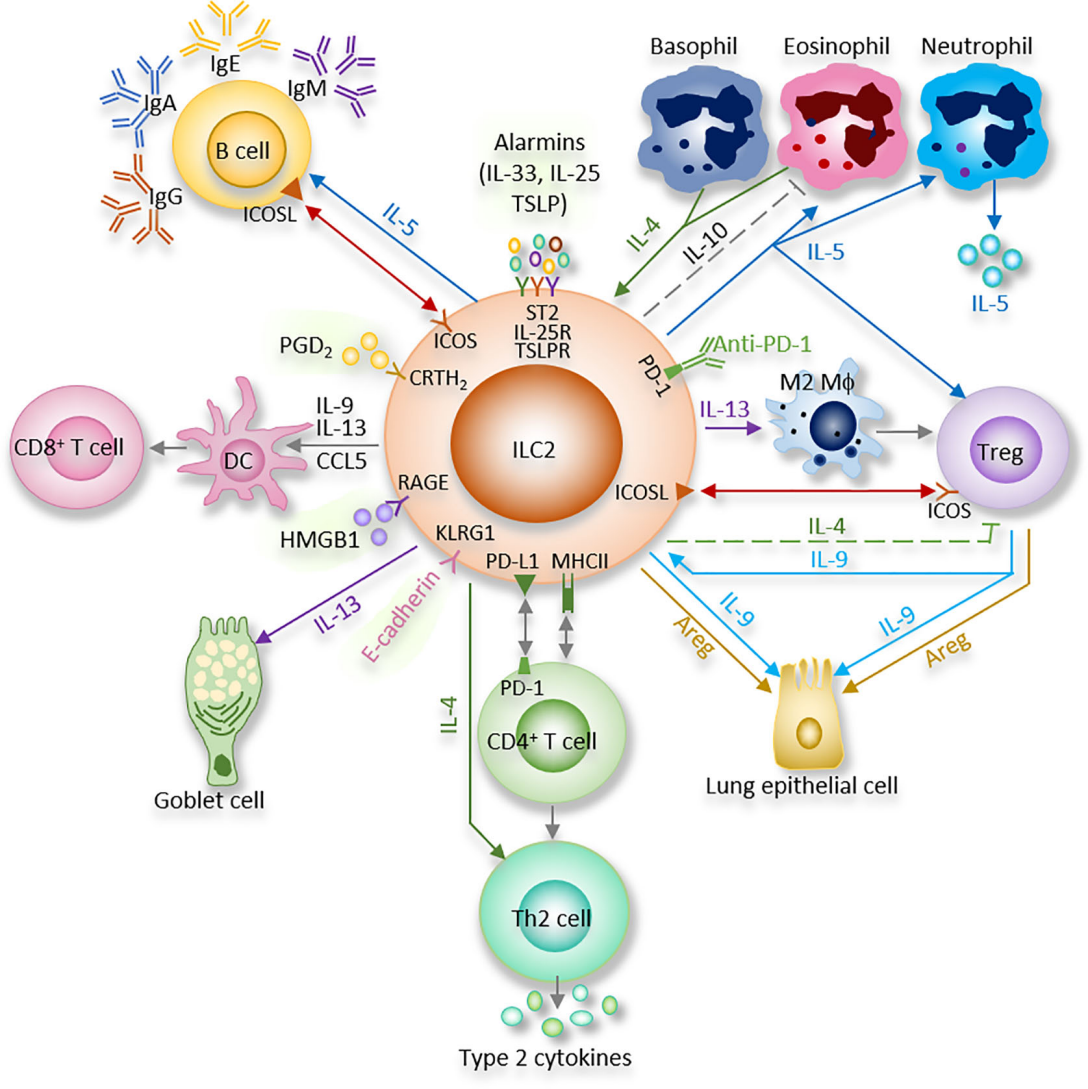
ILC2 interacts with other immune and non-immune cells through a variety of cytokines and cell surface mediators. (1) After activation with alarmins, ILC2 produce type II cytokines and mediators; (2) ILC2 interact with T cells *via* MHCII, CCL5, PD-1/PD-L1, OX40/OX40L, CD86, CD80, IL-4, IL-5, and IL-13; (3) ILC2 activate B cells to undergo isotype-switching, survival, self-renewal, and secrete antibodies *via* ICOS/ICOS-L, IL-5; (4) ILC2 stimulate Tregs *via* IL-5, IL-9, ICOS/ICOS-L, while ILC2-released IL-4 suppress Tregs. Tregs are capable of inhibiting ILC2; (5) ILC2 prime macrophages into a type 2 immune cell phenotype *via* IL-13; (6) Epithelial cells derived alarmins activate ILC2. ILC2-released IL-9 and Areg protect lung endothelial cells. (7) ILC2 increase eosinophils *via* IL-5, HMGB1. ILC2 inhibit eosinophils *via* IL-10. Eosinophil- or basophil-released IL-4 activate ILC2; (8) ILC2 activate DCs *via* IL-9, IL-13, HMGB1.

Many previous studies showed that “crosstalk” exist between T cells and ILC2 (**Figure 1**). For instance, ILC2 are the largest group of the cytokine-secreting leukocytes after ovalbumin or HDM treatment (59), and ILC2 activity is essential for the efficient differentiation of T_h_2 cells (29, 60–63). In Rag2^−/−^ mice, in which T cells and B cells are depleted due to *Rag* deficiency, the numbers of ILC2 also markedly decreased after helminth infection, indicating that T cells advance the survival of ILC2 (64). Epithelial cells derived cytokines and alarmins activate ILC2, which can be main producer of type 2 cytokines. Moreover, ILC2 can activate CD4^+^T cells either in the priming phase or during the effector phase since they present major histocompatibility complex class II (MHCII) (65, 66). IL-33–activated ILC2 enhances DCs migration into cancer tissues *via* C-C motif chemokine ligand 5 (CCL5) and further improve CD8^+^ T cell-mediated tumor immunity (67). Combination of anti-PD1 checkpoint blockade with rIL33 treatment collaborates to improve anti-tumor immunity by unleashing ILC2 activity (68). Activated ILC2 further facilitate the polarization of the anti-inflammatory M2 macrophages, which in turn stimulate Foxp3 regulatory T cells (Tregs) (69). Tregs are a subpopulation of T cells which modulate the adaptive immune responses through direct cell-cell interactions, as well as through the inhibitory functions of TGF-β and IL-10.

Through cytokines and interactions of ICOS with its ligand ICOS-L, ILC2 activate B cell to undergo isotype-switching, survival, and secrete IgG1 and IgE (64, 70, 71). IgE produced by B cells, together with type 2 cytokines released by T_h_2 cells and ILC2, lead to further activation of smooth muscle contraction, mucus production, granulocyte effector cells, and, which in turn, result in the encapsulation or expulsion of inflammatory stimuli (72). B cell-derived IgE is an important effector of type 2 immunity, and the recognition of allergens by IgE on mast cells is responsible for induction of the cardinal features of classic allergic responses, including anaphylactic shock (70). ILC2-derived IL-5 is an important growth factor that contributes to B1 cell self-renewal (73, 74). ILC2 sorted from mesenteric fat-associated lymphoid clusters are able to increase IgA production by peritoneal B cells *in vitro* (74).

Multiple indirect (cytokines) and direct (surface-bound molecules) mechanisms are involved in interactions between ILC2 and other cells as summarized below (**Figure 1**) (29, 75–77).

### IL-4

IL-4 is expressed by activated ILC2 (78, 79). ILC2-released IL-4 participates in blocking the expansion of allergen-specific Tregs, thus involving in food allergy (80). ILC2-released IL-4 is also able to polarize T_H_2 cells during helminth infection (81).

On the other hand, eosinophil- or basophil-released IL-4 is found to affect ILC2 by enhancing ILC2 lineage proliferation, function and stability (82, 83).

### IL-5

ILC2 can coordinate adaptive and innate immune functions through IL-5. IL-5 is critical for B cell function (74) and eosinophil homeostasis (**Figure 1**) (84, 85). When splenic B cells co-culture with mesenteric ILC2, IL-5 from ILC2 plays a pivotal role in the release of IgA from B cells (74). Besides, peripheral ILC2 in pulmonary, peritoneal cavity, and spleen are able to elevate the secretion of IgA, IgE, IgG1, and IgM by B cells in *ex vivo* co-cultures (86). Furthermore, upon NP-Ficoll (primes for a high affinity IgM anti-NP response) treatment *in vivo*, IgM produced by B cells is selectively initiated by lung ILC2 in an IL-5–dependent pattern (86). IL-5–producing ILC2 are also essential for the Th2 and Th9 cytokine responses against Trichinella spiralis infection (87). In addition, lung ICOS^+^ILC2 act a protective factor in a bleomycin model in an IL-5-dependent manner (88). Of note, the timing of IL-5 release by ILC2 seems important for the protective activity (88). Study showed that prostaglandin D_2_ (PGD_2_)-chemoattractant receptor-homologous molecule expressed on Th2 cells (CRTH2) signaling increases ILC2 and its production of IL-5, which promotes Tregs proliferation (89).

Our recent study discovered that high mobility group box 1 (HMGB1, a late mediator of sepsis) signals *via* receptor for advanced glycation end products (RAGE) to increase lung ILC2 by enhancing ILC2 proliferation and suppressing ILC2 death. The activated ILC2 increase type 2 cytokines production and eosinophil infiltration in the lungs, both of which improve haemorrhagic shock-induced acute lung injury (85). Lung ILC2 activated by IL-33 secrete a large number of IL-5, which further up-regulate neutrophil and its IL-5 production (90).

### IL-9

Price *et al*, reported in detail that ILC2 express IL-9 receptor (78). Using IL-9 reporter and subsequently IL-9 fate mapping mice, two studies delineated the autocrine signaling mechanism of IL-9 in ILC2 that enhances IL-13 and IL-5 release (**Figure 1**) (66, 91). In papain-induced pulmonary inflammation model, IL-9 was secreted for a short period by ILC2, then ILC2 changed to release IL-13 and IL-5. Furthermore, IL-33, but not IL-25, increased IL-9–producing ILC2 (91). Rauber *et al*, recently uncovered that IL-9 from ILC2 was necessary for Tregs activation and inflammation resolution in an arthritis model (92). HMGB1-activated ILC2 also secrete IL-9, which increase DCs (93). Moreover, our study discovered that ILC2-released IL-9 protects lung endothelial cells from pyroptosis by suppressing caspase-1 in a septic model (17).

IL-2 released by adaptive immune cells also play a crucial role in the IL-9 expression by ILC2, suggesting again the strong functional link between adaptive and innate lymphoid cells (94). Besides, IL-2 functions as a costimulator to ILC2 and promotes cell proliferation and survival by activating NF-κB pathway and gene transcription through p65 translocation (94). IL-9 and IL-2 work synergistically to direct ILC2 biology, and increased IL-9 production is related to an asthma-like phenotype in humans and mice highlighting the key role of these cytokines (95–98).

### IL-13

IL-13 also mediates the interaction between ILC2 and immune and non-immune cells (**Figure 1**). During infection of helminth in mice, IL-13 released from ILC2 is more abundant than that from Th2 cells for restricting worm expulsion and immune response (23, 64, 78). IL-13 from ILC2 can induce goblet cell hyperplasia as well as mucus secretion (99). Yet the precise mechanism of IL-13 receptors on pulmonary cells at different states remains to be fully elucidated.

Pulmonary IL-13^+^ ILC2 and CD4^+^ T cells cooperate to suppress Nippostrongylus brasiliensis (*Nb*) infection. Immune-damaged larvae have a severe morphological defect that is due to the increase of CD4^+^ T cells and IL-13^+^ ILC2, as well as the activation of M2 macrophages (100). Besides, alveolar macrophages can be primed by ILC2-derived IL-13 into a type 2 immune cell phenotype (101). DCs are stimulated by IL-13 to convert to a type 2 chemokine-secreting phenotype. ILC2-derived IL-13 is also able to mediate DCs migration from the lungs to the LNs, thus impacting the differentiation of T_H_2 cell (102). In addition, the number of IL-13^+^ ILC2 was reported to be markedly upregulated in patients with uncontrolled asthma, and it was significantly decreased when these patients had their symptoms controlled by treatment, suggesting an important role for ILC2-derived IL-13 in asthma (103). In conclusion, ILC2-derived IL-13 can initiate and affect innate and adaptive type 2 immune responses.

### Amphiregulin (Areg)

Areg is a member of the epidermal growth factors (EGF) family and acts *via* the EGF receptor (EGFR) (104). Both hematopoietic and non-hematopoietic cells in the lung present EGFR (105). ILC2 are a major cellular producer of Areg after activation with IL-33. ILC2-derived Areg is a critical component of effective pulmonary wound healing during influenza infection and restoring epithelial integrity and lung function (20). The initiation of mucus secretion and wound healing can prevent or ameliorate some respiratory diseases, although enhanced and excessive mucus may play a detrimental role in diseases, such as asthma (106, 107). Pulmonary Tregs are also capable of producing Areg without TCR signaling (**Figure 1**) (108). Thus, innate Areg released by ILC2 and Tregs is of high importance to restore tissue homeostasis and wound healing after pulmonary infection.

### ICOS/ICOS-Ligand Interaction

Studies on helminth expulsion revealed ILC2-Tregs crosstalk (109). Tregs and ILC2 colocalize to similar regions within the lung tissues and visceral adipose tissue under homeostatic and inflammatory conditions (**Figure 1**) (31). Of note, ICOS^+^ Tregs and ICOS-L^+^ ILC2 were reported to accumulate in tissues after *Nb* infection or IL-33 administration, while Tregs accumulation in ICOS-L knock-out mice or after administration with neutralizing monoclonal antibody against ICOS-L was reduced, indicating that ICOS-L^+^ ILC2 could improve Tregs expansion, thus establishing a pathway for Tregs to cooperate with ILC2 (31). On the other hand, Tregs are capable of inhibiting ILC2 to restrict allergic airway inflammation (110). Besides, ICOS/ICOS-L interaction on ILC2 affects STAT5 signaling, activating ILC2 function and proliferation in an allergic model (111).

### PD-1/PD-L1 Interaction

ILC2 constitutively express the checkpoint inhibitor molecule programmed death-ligand 1 (PD-L1), which has been discovered to activate CD4^+^ Th2 cell responses during type 2 pulmonary responses (**Figure 1**) (30). Conditional knockout of PD-L1 on ILC2 disrupted cytokine production and early Th2 polarization, resulting in delayed worm expulsion during infection with the gastrointestinal helminth *Nb* (30). Nevertheless, ILC2 can also express PD-1, which was reported to be upregulated on activated ILC2, and depletion of these PD-1^+^ ILC2 resolves papain-induced lung inflammation (112).

### E-Cadherin/KLRG1 Interaction

E-cadherin, a cell adhesion molecule, interacts with the mature ILC2 marker, killer-cell lectin like receptor G1 (KLRG1). The finding that E-cadherin-KLRG1 ligation on human ILC2 reveals a significant decrease in GATA3 expression and type 2 cytokine (such as IL-5 and IL-13) release and the discovery that E-cadherin expression is suppressed in keratinocytes propose that inhibited E-cadherin expression may activate ILC2, promoting atopic dermatitis (AD) immunopathogenesis (28).

### Other Interactions

OX40 ligand (OX40L) expression on ILC2 can be enhanced by IL-33. OX40-OX40L ligation has been reported to increase Th2 cell survival and number (32, 113), thus promoting adaptive immunity (75).

A molecularly distinct subset of lung ILC2 can secrete IL-10 and suppress some pro-inflammatory genes. IL-2, IL-4, IL-27, IL-10, and NMU stimulate IL-10 production from ILC2 and are associated with decreased eosinophil recruitment to the lung, indicating that ILC2 have anti-inflammatory functions similar to Tregs (**Figures 1** and **3**) (26, 114).

**Figure 2:**
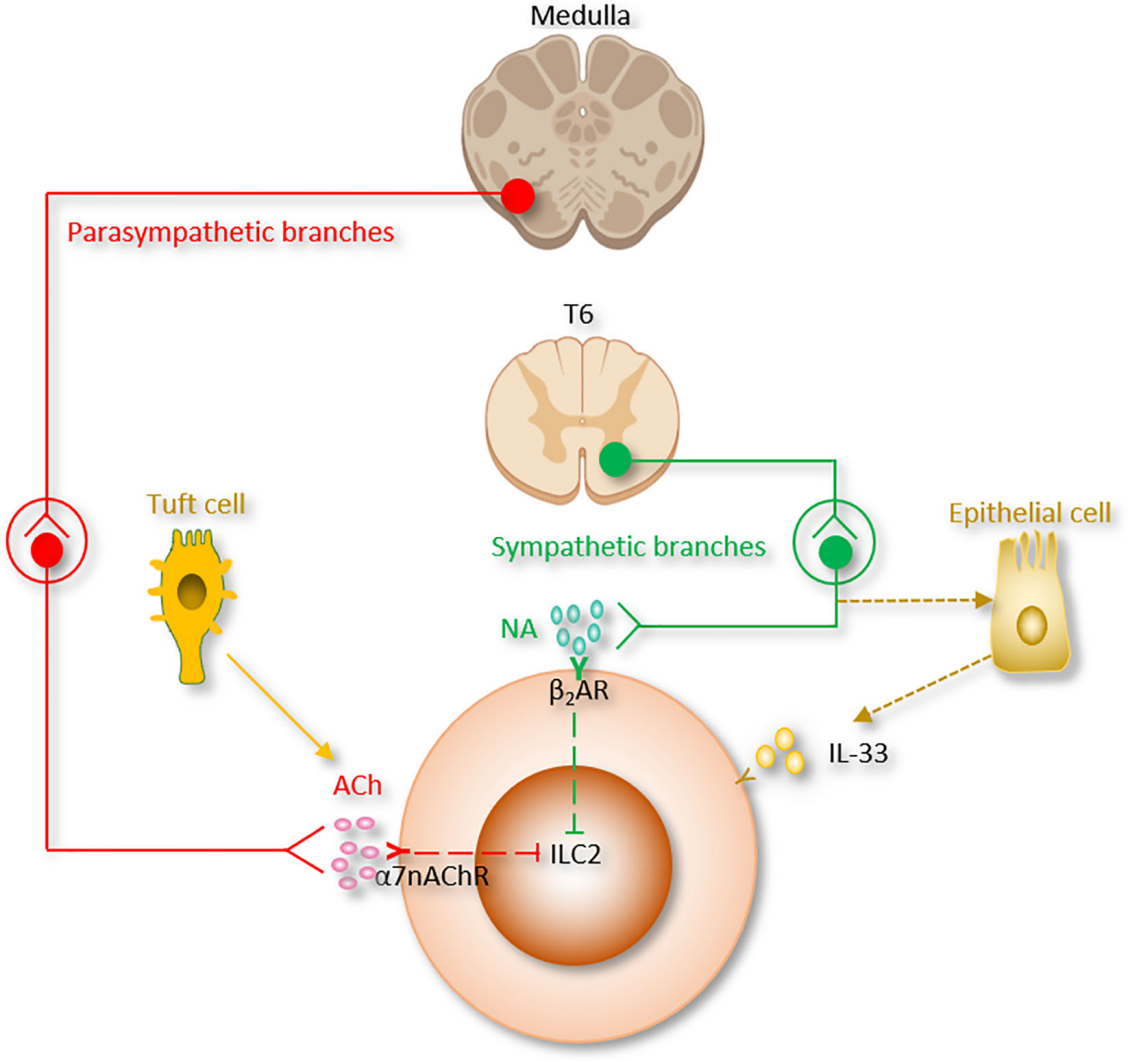
The autonomic nervous system regulation of ILC2 function. Ach, Acetylcholine; α7nAChR, α7 nicotinic acetylcholine receptor; β_2_AR, β_2_-adrenergic receptor; NA, Norepinephrine.

**Figure 3:**
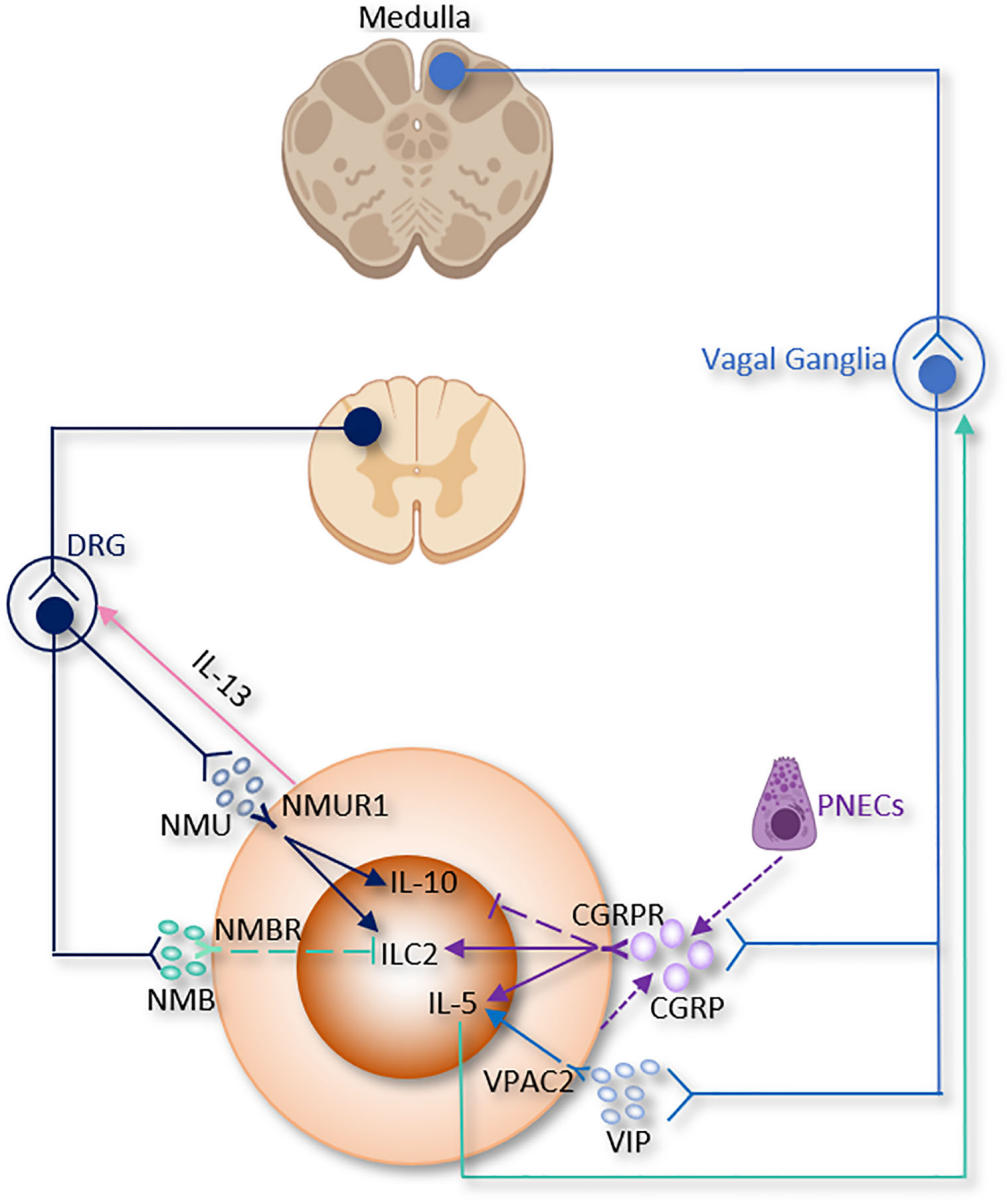
The somatic nervous system regulation of ILC2 function. CGRP, α-calcitonin gene-related peptide; DRG, Dorsal root ganglion; NMB, neuromedin B; NMU, neuromedin U; PNECs, Pulmonary neuroendocrine cells; VIP, Vasoactive Intestinal Peptide.

The expression of CD86, CD80, and MHCII by mouse ILC2 is also involved in ILC2 interactions with CD4^+^ T cells;

MHCII^+^ ILC2 can drive the differentiation of naive CD4^+^ T cells into T_h_2 *in vitro*, whereas MHCII-deficient ILC2 upregulate T_h_2 cell-driven helminth expulsion *in vivo* (29, 75).

## Pulmonary Nervous System and its Regulatory Function

Lung is densely innervated by peripheral nervous system (PNS), which is divided into motor and somatosensory nervous systems (**Figure 2**).

### Motor Nervous System

The motor nervous system consists of the autonomic (sympathetic, parasympathetic, and enteric) and somatic branches.

Autonomic nervous system serves to regulate involuntary functions. Sympathetic branch comes from the upper six thoracic spinal cord segments; the synapse together with the sympathetic ganglia, and postganglionic fibers then innervate the airways. The sympathetic nervous system participates in the body response to stress and regulates bronchodilation and the production of mucous.

The cholinergic parasympathetic branch comes from the vagal nuclei of the medulla; the superior and recurrent laryngeal vagal nerves synapse at the parasympathetic ganglia to innervate the lung (115). The parasympathetic branch is mainly responsible for keeping homeostasis (116). It regulates bronchoconstriction, carbon dioxide and oxygen levels, as well as neural reflexes including coughing.

### Somatosensory Nervous System

The somatosensory nervous system delivers sensory stimuli, such as proprioception, touch, and pain. Somatosensory neurons are further divided into pruriceptors and nociceptors responsible for sensing itch-inducing or noxious stimuli, respectively. These neurons are important because their activation is closely related to inflammation and immunity (**Figure 2**).

All opponents of the PNS play a critical role in orchestrating immunity, inflammation, and tissue repair at host barrier tissues in response to stimuli and stressors. Primary evidence that neurotransmitters may regulate immune responses was that their release from nerves could lead to signaling through the surface receptors of lymphocyte cell (117). Leukocytes have receptors for the neurotransmitters such as dopamine, serotonin and glutamate (117), and also produce neurotransmitters that work as paracrine or autocrine signals (118). The neuronal reflex senses peripheral inflammation and coordinates the host response to injury and/or infection, regulating events within the initiation of inflammation (48, 119). Lung is heavily populated by resident immune cells, such as macrophages, DCs, γδ T cells mast cells and ILCs. It allows fast, integrated reactions to pathogens and noxious stimuli (120).

### Sympathetic Nervous System

Sympathetic nervous system helps the center nervous system to control innate immune responses between antiviral and pro-inflammatory actions (**Figure 2**) (121, 122).

The nerves of the sympathetic nervous system distribute the neurotransmitter catecholamines into tissue microenvironments in which immune response gene transcription occurs, including all lymphoid organs, most musculoskeletal structures and visceral organs, and the vasculature and perivascular tissues (123).

Recent reports have revealed a sympathetic nervous system-mediated steering of innate immune response programs, which include enhanced transcription of pro-inflammatory cytokine genes (such as *Il6*, *tnf*, and *Il1β*) (121, 124) and inhibition of type I IFN-mediated antiviral responses (122).

Stimulation of the sympathetic nervous system has also been shown to change the production and trafficking of innate immune cells, for instance, through the upregulation of myelopoiesis and the mobilization of monocytes, splenic neutrophils, natural killer cells, and haematopoietic stem cells (123). A current study showed that the sympathetic nervous system stimulates IL-33 and then ILC2 in adipose tissue. Cold exposure stimulates IL-33 expression, ILC2 and eosinophils in adipose tissue. Furthermore, sympathetic denervation induced by 6-hydroxydopamine (6-OHDA) cancels this effect (125).

Catecholamines are monoamine neurotransmitters which are mainly released by the postganglionic fibers of the sympathetic nervous system and the chromaffin cells of the adrenal medulla. Included among catecholamines are dopamine, epinephrine (adrenaline), and norepinephrine (noradrenaline) (**Table 1** and **Figure 2**). Release of the epinephrine and norepinephrine from the adrenal glands and adrenergic nerves is part of the fight-or-flight response.

**Table 1:** Sources, receptors on ILC2, and relationships with ILC2 of several neurotransmitters.

Neurotransmitters		Sources		Relevant receptors on ILC2	Relationships with ILC2
Catecholamines	Epinephrine (Adrenaline)	Autonomic (Involuntary)	Sympathetic nervous system;	β_2_AR	Adrenergic neurons colocalize with ILC2; β_2_AR agonist administration impairs ILC2 responses and reduces inflammation; Mediator of the “anti-inflammatory reflex”;
	Norepinephrine				
Acetylcholine			Parasympathetic nervous system; Also released by tuft cells;	α7nAChR	α7nAChR agonist administration inhibits the proliferation of ILC2, but does not alter the death of ILC2; α7nAChR agonist administration inhibits type 2 cytokine production from ILC2 and ameliorates ILC2-mediated lung inflammation; Mediator of the “anti-inflammatory reflex”;
CGRP		Somatic (Voluntary, afferent and efferent neurons)	Sensory neurons; Also released by PNECs and ILC2;	RAMP1 and CALCRL	CGRP-secreting PNECs locate in close proximity to ILC2 near airway branch points; ILC2 express both CGRP and its receptor CGRPR; CGRP stimulates ILC2 proliferation; CGRP suppresses KLRG1^+^ILC2s proliferation but promotes IL-5 expression; CGRP alone does not increase cytokine production from ILC2, a combination of (NMU + IL-33 + CGRP) stimulates IL-5 but limits IL-13 production and ILC2 proliferation;
VIP			Sensory neurons;	VPAC1 and VPAC2	VIP stimulates IL-5 from ILC2, ILC2-derived IL-5 activates nociceptors on sensory neurons and upregulates the release of VIP, which in return acts *via* VPAC2 and leads ILC2 and subsequently T cells to release more IL-5 and thereby forming a type 2 inflammatory positive feedback loop mainly based on the neuro-immune axis;
NMU			Sensory neurons (released by cholinergic sensory neurons originating from DRG); Also secreted by some APCs (including monocytes, B cells, and dendritic cells);	NMUR1	NMU-expressing neurons locate in close vicinity to ILC2; NMU elevates ILC2 proliferation; Stimulation of ILC2 with NMU leads to strong and immediate production of tissue protection and innate inflammatory cytokines in a NMUR1-dependent manner; NMU increases IL-10 production in activated ILC2, IL-10 further stimulates IL-10 production in ILC2 through a positive feedback loop; ILC2 activated by NMU increase the number of lung eosinophils and mast cells; IL-13 enhance NMU production in DRG neurons, thus indicating the existence of a reciprocal neuron–ILC2 regulatory loop *via* ILC2-derived IL-13 and neuronal NMU expression;
NMB		CNS (olfactory bulb, dentate gyrus, amygdala, basal ganglia, brainstem); PNS (Gastrointestinal tract; Trigeminal and dorsal root ganglia (DRG));		NMBR	Treatment with NMB inhibits ILC2 responses, eosinophilia and mucus production; Basophils prime ILC2 for NMB-mediated inhibition;

Catecholamines exert their effects *via* two classes of adrenergic receptors, α and β. Both groups could be functionally divided into subgroups (α_1_ and α_2_; β_1_, β_2_, and β_3_).

Norepinephrine regulates leukocyte gene expression through β-adrenergic receptors (123). β-adrenergic receptor is expressed on most immune cells, such as B cells, T cells, and other innate cells (40, 126–128). It was initially thought to regulate adaptive immune responses by suppressing the expression of TH1-type genes, such as *Il12β* and *Ifnγ*, and activating the transcription of TH2-type cytokine genes, i.e. *Il4* and *Il5* (129–131).

Interestingly, β_2_AR has an inhibitory effect on innate immune responses. β_2_-agonists suppress cardiodepressant and inflammatory factors, including HMGB1 and TNF. Recently, ILC2 from the lung and the gut-related tissues (small intestinal lamina propria, colonic lamina propria and mesenteric lymph nodes) were found to express β_2_AR. ILC2 were also shown to colocalize with adrenergic neurons in the mouse intestine (40).

β_2_AR deficiency led to enhanced ILC2 proliferation and subsequent type 2 cytokine production in lung and intestinal tissues after infection with *Nb*. Lung eosinophilia was observed following enhanced IL-5 production from ILC2 in β_2_AR-deficient mice. On the other aspect, β_2_AR agonist administration disrupted ILC2 responses and suppressed inflammation *in vivo*. By using conditional β_2_AR-deficient mice, or by transferring ILC2 progenitors from wild-type mice or β_2_AR-deficient mice into ILC-deficient mice, the group generated ILC2-specific β_2_AR-deficient mice and ensured that the β_2_AR negatively controls ILC2 and type 2 inflammation (40). This study provides another evidence of neuronal regulatory circuit that regulates ILC2-dependent type 2 inflammation. β_2_AR-agonists are the most effective medications for the treatment of asthma. β_2_AR-mediated ILC2 regulation could be one of the pathways of β_2_AR-agonists effect in asthma (33, 38, 44, 110, 132, 133). On the other hand, β_2_AR is the first adrenergic receptor documented to participate in the “anti-inflammatory reflex” of the parasympathetic system which will be discussed in the next section (**Table 1** and **Figure 2**).

### Parasympathetic Nervous System

Parasympathetic and sympathetic systems are usually considered to work in opposition to maintain physiological homeostasis. While current literatures suggested that both branches work together to restrain systemic inflammation in life-threatening illnesses, including arthritis, inflammatory bowel disease, sepsis and endotoxemia (46, 48, 134–138).

Nerve fibers of the parasympathetic nervous system arise from three primary areas: cranial nerves (facial nerve, oculomotor nerve, and glossopharyngeal nerve), vagus nerve, and pelvic splanchnic nerves (three spinal nerves in the sacrum, S2-4).

The parasympathetic nervous system mainly utilizes acetylcholine (ACh) as its neurotransmitter (**Table 1** and **Figure 2**). Tuft cells, capable of secreting the ILC2 activator IL-25, also secrete Ach (139, 140). The ACh has two kinds of receptors, the nicotinic and muscarinic cholinergic receptors. α7 nicotinic acetylcholine receptor (α7nAChR), one of the nicotinic acetylcholine receptors, is expressed by ILC2 at steady state, and this expression is further increased following alarmin-induced activation such as IL-33 (44). The α7nAChR is also present on B cells (141), T cells (142, 143), macrophages (144) and ILC3 (41).

α7nAChR^-/-^ mice were more susceptible to severe lung injury and higher mortality than α7nAChR^+/+^ mice. Increased α7nAChR^+^ alveolar neutrophils and macrophages were observed in the mice injured lungs. The immunomodulatory cholinergic α7nAChR pathway of alveolar neutrophils and macrophages alleviated E. coli- and LPS-induced acute lung injury by inhibiting transalveolar neutrophil migration and chemokine production.

It was reported that the expression of HMGB1 protein was suppressed by α7nAChR agonist nicotine and the survival of post-sepsis acute lung injury was improved (145). In addition, administration of α7nAChR agonist inhibits type 2 cytokine production from ILC2 and ameliorates ILC2-mediated lung inflammation induced *via* IL-33 stimulation or Alternaria alternata inhalation. Mechanistically, α7nAChR agonist is reported to inhibit cellular markers for proliferation in ILC2 (Ki67, NF-κB and GATA3 signaling pathways) (27, 44, 146, 147) (**Table 1**). These findings indicate that α7nAChR may be a potential therapeutic target for acute lung injury (120).

On the other aspect, Bcl-2, an anti-apoptotic factor of ILC2, was unchanged by α7nAChR agonist treatment. These studies indicate that parasympathetic nervous system modulates ILC2 proliferation, but not death. The suppressive effects of α7nAChR on ILC2 may serve as the mechanism underlying the observed reduced pulmonary allergic inflammation induced by nicotine treatment (148). Moreover, in cancer immunity, nicotine treatment stimulates tumor growth by suppressing apoptosis and promoting cell proliferation (149–152).

### Anti-Inflammatory Reflex

The vagus nerve is the main parasympathetic nerve connecting between the central nervous system and peripheral organs (128, 153). Pharmacological or electrical stimulation of the vagus nerve can restrain the systemic inflammation response, organ damage, and mortality in different experimental hemorrhage and resuscitation (43), pancreatitis (154), ischemia and reperfusion (43, 155, 156), colitis (157), endotoxemia (42, 155, 158, 159) and sepsis (145, 160).

Mechanically, the motor and sensory vagus nerve form a complex neural reflex circuit termed the “anti-inflammatory reflex” which control spleen cytokine production through splenic nerve (42, 161–166). ACh released by the vagus nerve in the celiac mesenteric ganglia stimulates postsynaptic α7nAChR of splenic nerve (46). The adrenergic splenic nerve release norepinephrine to activate a discrete subset of spleen lymphocytes *via* β_2_AR. Activated lymphocytes then release Ach (48, 167). Lymphocyte-derived Ach downregulates macrophage cytokine release, and switches them toward a tissue-protective, M2 anti-inflammatory phenotype. α7nAChR mediates Ach-induced signal transduction in macrophages and monocytes (144). α7nAChR inhibits the inflammasome activity (168), enhances the JAK2-STAT3 pathway (169), stabilizes mitochondrial membranes, and suppresses the nuclear translocation of NF-κB (119, 145, 168–170).

The eventual influence of inflammatory reflex on the spleen is the inhibition of cytokine release by spleen macrophages, which produce over 90% of the IL-1 and TNF during acute endotoxemia (159, 167). The anti-inflammatory reflex is a special instance of a functional network between the efferent parasympathetic vagus nerve, the splenic nerve (termed sympathetic) and T cells relaying neural signals. In depicting this cooperation, the use of the classical sympathetic-versus-parasympathetic neuronal designation should be modified.

Indirect studies suggested that anti-inflammatory reflex is involved in the regulation of ILC. Dalli *et al*, reported that dissection of the right vagus downregulated the number of peritoneal ILC3 and changed peritoneal macrophage responses. Right vagotomy led to decreased peritoneal levels of pro-resolving mediators, which include the protective immunoresolvent protectin conjugates in tissue regeneration 1 (PCTR1), as well as increased inflammation-initiating eicosanoids. Ach restored the PCTR1 production from ILC3. Treatment of PCTR1 or ILC3 repaired tissue and ameliorated *E. coli* infections in vagotomized mice (41). Another group studied the regulation of ILC2 using coding α7nAChR (Chrna7) knockout mice, pulmonary C fibre (PCF, which releases ACh and neuropeptides) degeneration mice, and vagotomized mice. Knockout of Chrna7 enhanced resident ILC2s and trafficking iILC2s in the lung, worsened allergic inflammation. However, PCF degeneration and vagotomy significantly reduced these two types of ILC2 and attenuated asthma responses (171). Although there is no direct evidence suggests that “anti-inflammatory reflex” regulates ILC2, it is promising to investigate since the number of ILC2 is significantly increased in spleen during inflammation or infection (172).

In sum, parasympathetic nervous system participates in the pathogenesis of various diseases, with a different role in each disease.

### Sensory Neurons

The lung is innervated by a dense network of sensory neurons that mainly comes from vagal afferents whose cell bodies reside in the vagal ganglia (jugular and nodose ganglia); while other sensory nerve innervation originates from the dorsal root ganglion (DRG) (**Figure 3**) (115, 173–175). Nociceptive receptors are richly expressed in sensory neurons endings, which are abundantly present in the lung parenchyma and near the airways; this poises them to act as the first wall for host defense and these neurons interact directly with inflammatory stimuli such as ATP, pathogens, allergens, protons, heat, mechanical injury and chemical irritants like immune cells such as APCs, macrophages, and other phagocytes (116, 176–178).

Sensory neuronal action potentials evoked by this interaction are then transmitted into the CNS within milliseconds of the detection of inflammation or invasion. This action-potential signaling mechanism is significantly faster than immune cell. Once activated, nociceptive receptors induce coughing, pain and bronchoconstriction (173, 178–180). Neuropeptides emanating from nociceptor nerve terminals also participate in the nociceptors crosstalk with immune cells (173, 181–183).

Excitation of nociceptors increases the release of multiple neuropeptides, such as substance P, VIP and CGRP which regulate both innate and adaptive immune cells (184) (**Table 1** and **Figure 3**).

### CGRP

CGRP, encoded by *Calca*, is a member of the calcitonin family peptides that not only secreted by peripheral nociceptive neurons but also found in central neurons (45, 185). CGRP binds to a heteromeric receptor composed of a receptor activity-modifying protein (RAMP1) and a G-protein coupled receptor termed CALCRL. CGRP *via* these receptors stimulates AC, which results in cAMP and PKA pathway activation and leads to the phosphorylation of several downstream pathways including NOS, MAPK, and CREB pathways (186).

In skin bacterial infection, lymph node hypertrophy and TNF-α production are found to be suppressed by CGRP (183, 187). CGRP levels markedly increased in the bronchoalveolar lavage fluid (BALF) after Staphylococcus aureus infection. S. aureus also increases cultured neuronal production of CGRP *in vitro*. CGRP could alleviate the symptoms of S. aureus-induced pneumonia by suppressing TNF-α, CXCL1, γδ T cells and neutrophils (188).

A recent study discovered the relationship between CGRP and pulmonary neuroendocrine cells (PNECs), which comprise ~1% of the airway cell population (189, 190). PNECs (locate in close proximity to ILC2 near airway branch points) could secrete CGRP, which aggravates allergen-induced asthma in mice by stimulating ILC2 proliferation and the secretion of IL-5 from ILC2 (133). On the other hand, *Il5* ^hi^ILC2 produce both CGRP and its receptor CGRPR following *Nb* infection. CGRP treatment alone does not increase cytokine production from ILC2, a combination of neuromedin U (NMU) + IL-33 with CGRP stimulates IL-5 but limits IL-13 production and ILC2 proliferation. Worm expulsion and ILC2 responses are augmented without CGRP signaling (39). Interestingly, Xu et al, reported that OVA-induced inflammation increased the expression of *Calca* in KLRG1^+^ILC2. CGRP suppressed KLRG1^+^ILC2s proliferation but promoted IL-5 expression (191). Collectively, these paradoxes point to both pro- and anti-inflammatory properties of CGRP on immune responses in the lung and warrant further investigation (**Table 1** and **Figure 3**).

### VIP

The neuropeptide VIP also involves in the regulation of ILC2. It has been firstly characterized as a polypeptide isolated from the small intestine with multiple impacts on different systems such as respiratory and cardiovascular systems (192). VIP can be perceived by VIP receptor type 1 (VPAC1) or VIP receptor type 2 (VPAC2), which are differentially regulated according to cell type and activity conditions (193, 194). Similar to CGRP, VIP enhances the AC/cAMP/PKA pathway and phospholipase C, which causes the accumulation of intracellular Ca^2+^ (195).

Of note, pulmonary and intestinal ILC2 express VPAC1 and VPAC2 and produce IL-5 when they are incubated with IL-7 and VIP- or VPAC2-specific agonist (84). Talbot *et al*, discovered a critical relationship between ILC2, VIP, T cells, and nociceptive neurons (182). Reciprocally, ILC2-derived IL-5 activates nociceptors on afferent neurons and upregulates the release of VIP, which in return acts *via* VPAC2 and leads ILC2 and subsequently T cells to release more IL-5 and thereby forming a type 2 inflammatory positive feedback loop mainly based on the neuro-immune axis (**Table 1** and **Figure 3**) (182). Since the levels of blood eosinophils and type 2 cytokine release from ILC2 are regulated by circadian rhythm and food intake, this suggests that VIP might influence blood eosinophils *via* upregulation of ILC2 (84).

### NMU

NMU is a neuropeptide mainly released by cholinergic sensory neurons originating from DRG, but not parasympathetic neurons in the vagal ganglion (196). The initial biological functions ascribed to NMU were food intake and body weight reduction, smooth muscle contraction of the uterus, pronociceptive effects promotion and circadian rhythm regulation (197, 198). In addition, NMU is also occasionally secreted by some APCs, including monocytes, B cells, and dendritic cells (199). Thus, it is suggested to play an important role in the regulation of adaptive and innate immunity. In an allergen-induced asthma model, airway eosinophilia was shown to decrease in *nmu* ^-/-^ mice. NMU directly stimulated extracellular/signal-regulated kinase phosphorylation and Ca^2+^ mobilization. NMU also induced cell adhesion to components of the extracellular matrix, and chemotaxis *in vitro* (200).

Recent studies reported that NMU from lamina propria play a regulatory role in mice type 2 innate immunity through binding to the neuromedin U receptor 1 (Nmur1), which is selectively enriched in ILC2. Consistent with this idea, NMU-expressing neurons have been discovered in close vicinity to ILC2 in the lungs (19, 37, 38). Lung ILC2 present NMUR1 at steady state and upon IL-25 stimulation, however, NMUR1 was inhibited upon IL-33 exposure (38).

In a mice model of worm infection in the lungs and intestine, stimulation of ILC2 with NMU led to strong and immediate production of tissue protection and innate inflammatory cytokines in a NMUR1-dependent manner, thereby alleviating worm burden (37).

In a model of airway allergy, ILC2 were activated by NMU *in vitro*, and *in vivo* co-treatment of NMU with IL-25 significantly increased lung histopathology. Disruption of NMU-NMUR1 pathway decreased ILC2 number and effector function, and changed transcriptional programs following *in vivo* allergen exposure (38). NMU elevates pulmonary ILC2 proliferation and a selectively potent secretion of innate IL-5, IL-13, and amphiregulin (19, 37, 38). Furthermore, ILC2 activated by NMU increase the number of lung eosinophils and mast cells, thus alleviating antihelminth responses (19, 37, 38). Interestingly, IL-13 enhance NMU production in DRG neurons, thus indicating the existence of a reciprocal neuron–ILC2 regulatory loop *via* ILC2-derived IL-13 and neuronal NMU expression (38). IL-10, primarily secreted by Tregs, was increased by NMU in activated intestinal ILC2. IL-10 further stimulated IL-10 production in ILC2 through a positive feedback loop (**Table 1** and **Figure 3**) (114). These findings suggested that NMU treatment enhance inflammation-induced damage in the lungs and pointed to a double-edged sword of NMU-NMUR1 signaling.

### NMB

NMB belongs to the neuromedin family that includes NMB, NMC, NMK, NML, NMN, NMU and NMS (201, 202). It is expressed widely in the CNS (olfactory bulb, dentate gyrus, amygdala, basal ganglia, and brainstem) and the PNS (gastrointestinal tract, trigeminal and dorsal root ganglia (DRG)) (203, 204).

NMB controls cell growth, blood glucose, body temperature, emotion, energy homeostasis, exocrine and endocrine secretion, food intake, grooming and scratching, nociception and smooth muscle contraction. Inclan-Rico, Juan M *et al*, found that administration of NMB suppressed ILC2 responses *via* NMU receptor (NMBR), eosinophilia and mucus production after *Nb* infection in the lung. In consistent with *in vivo* results, *in vitro* treatment of NMB inhibited the growth of sorted ILC2 (**Table 1** and **Figure 3**). Of note, ILC2 sorted from basophil-depleted mice were unchanged by NMB stimulation, indicating that basophils are indispensable for the inhibitory effects of NMB on ILC2 (50).

## Promising Directions for Research on the Neural Regulation of ILC2 in the Lung

The discoveries of neural control of ILC2 have added a new dimension to neuroimmunity. All previously known findings of ILC2 in the lung could be re-examined from this perspective.

Previous studies on the lung ILC2 have been mainly performed in models of allergic disease (205–208), helminth infection (16, 50, 209, 210), and septic lung injury (17, 85, 211, 212). Multiple neural pathways have been reported to be involved in these disease models (4, 213, 214). Although recent studies have found some clues, the relationship between nervous system and ILC2 still remains contradictory and inconclusive. For example, CGRP shows opposite effects on different ILC2 subtypes. NMU is able to enhance the pro-inflammatory function of ILC2 as well as its anti-inflammatory function. Does the nervous system or neural mediators play ameliorating or worsening roles in these diseases by regulating ILC2? If future studies can prove this hypothesis, then ILC2 will not only be a bridge between innate and adaptive immunity, but also between the nervous system and the immune system.

The second promising research direction will be the effects of neural-regulated ILC2 on nervous system. Type I cytokines and their receptors (such as IL-1, IL-6, and TNFs) are expressed widely in CNS cells and are important for the development and function of the CNS (58). However, the impacts of ILC2-released type II cytokines and mediators on nervous system remains to be elucidated. Currently, a reciprocal DRG–ILC2 regulatory loop *via* ILC2-derived IL-13 and neuronal NMU expression has been found (38). Besides, ILC2-derived IL-5 activates nociceptors on afferent neurons and upregulates the release of VIP, which in return, acting *via* VPAC2 leads to ILC2 and T cells to release more IL-5 and, thereby, forming a type 2 inflammatory positive feedback loop (182). It would be important to explore unidentified neuron-ILC2 positive/negative regulatory loops.

The third area of interest will be the neural regulation of ILC1 and ILC3. Recent studies have shown that three ILCs are functionally plastic. For instance, plastic iILC2 can coproduce both type-2 cytokines and the ILC3-characteristic cytokine (IL-17A) (215). Under certain conditions, c-Kit^+^ILC2 can convert to ILC3-like cells (216). Besides, IL-12 and IL-18 converted ILC2 into ILC1 in patients with chronic obstructive pulmonary disease (COPD) (217). Does neuromodulation affect the interconversion of ILC2 and two other cell subtypes? If these plastic properties can be elucidated, we can gain a comprehensive understanding of the relationship between ILC as a cell type and the nervous system.

Least but not last, these results have great therapeutic implications for precision medicine. For example, NMUR1 is selectively expressed by ILC2, while receptors for classical ILC2 activators, i.e. IL-25, IL-33, and TSLP, are widely expressed by various cell types (19, 37, 38). Meanwhile, researchers have developed many methods to selectively stimulate and inhibit neurons (218, 219). Combining these advances will allow us to identify more effective clinical targets.

## Conclusion

Emerging evidence from *in vivo* animal models, human studies, and *in vitro* experiments indicates that neuropeptides and neurotransmitters released from various neurons and non-neuronal cells are critical in regulation of immune responses in different tissues including the lung. This review article provides a comprehensive overview of the effects of novel neural mediators and pathways on ILC2 and underlying mechanisms as well as the insights into the direct and indirect interactions between ILC2 and other immune cells, highlighting ILC2 as the bridge between innate and adaptive immunity. However, the research in neuro-immune area is, in general, in a premature status, and numerous questions remain to be addressed. For examples, the most of signaling pathways that mediate neural regulation of ILC2 are yet clear; and the mechanisms, by which ILC2 selectively respond to neutral and non-neural signaling need to be elucidated as well. In addition, translational and clinical investigations are required to promote the application resulted from the studies in this area.

## Author Contributions

WC collected the data and drafted the manuscript. WC, QS, and JF conceived and designed the study. JF reviewed and finalized the manuscript. All authors contributed to the article and approved the submitted version.

## Funding

This work was supported by the USA National Institutes of Health Grant R01-HL-079669 (JF), USA National Institutes of Health Grant R01HL076179 (JF), USA National Institutes of Health Grant R01-HL-139547 (JF), VA Merit Award 1I01BX002729 (JF), and VA BLR&D Award 1IK6BX004211 (JF).

## Conflict of Interest

The authors declare that the research was conducted in the absence of any commercial or financial relationships that could be construed as a potential conflict of interest.

## Abbreviations

α7nAChR, α7 nicotinic acetylcholine receptor; Ach, Acetylcholine; AD, Atopic Dermatitis; Areg, Amphiregulin; CGRP, α-Calcitonin Gene-Related Peptide; CRTH2, Chemoattractant Receptor-homologous molecule expressed on Th2 cells; DCs, Dendritic Cells; PGD2, Prostaglandin D2; EGF, Epidermal Growth Factors; EGFR, EGF Receptor; HMGB1, High Mobility Group Box 1; ICOS, Inducible Costimulator; ILCs, Innate Lymphoid Cells; ILC2, Group 2 Innate Lymphoid Cells; KLRG1, Killer-cell Lectin Like Receptor G1; NMU, Neuromedin U; NMB, Neuromedin B; Nb, Nippostrongylus brasiliensis; PCTR1, Protectin Conjugates in Tissue Regeneration 1; PD-L1, Programmed Death-Ligand 1; PNECs, Pulmonary Neuroendocrine cells; PNS, Peripheral Nervous System; RAGE, Receptor for Advanced Glycation End products; RAMP1, Receptor Activity-Modifying Protein; TLRs, Toll-Like Receptors; Tregs, Regulatory T Cells; TSLP, Thymic Stromal Lymphopoietin; VIP, Vasoactive Intestinal Peptide.
